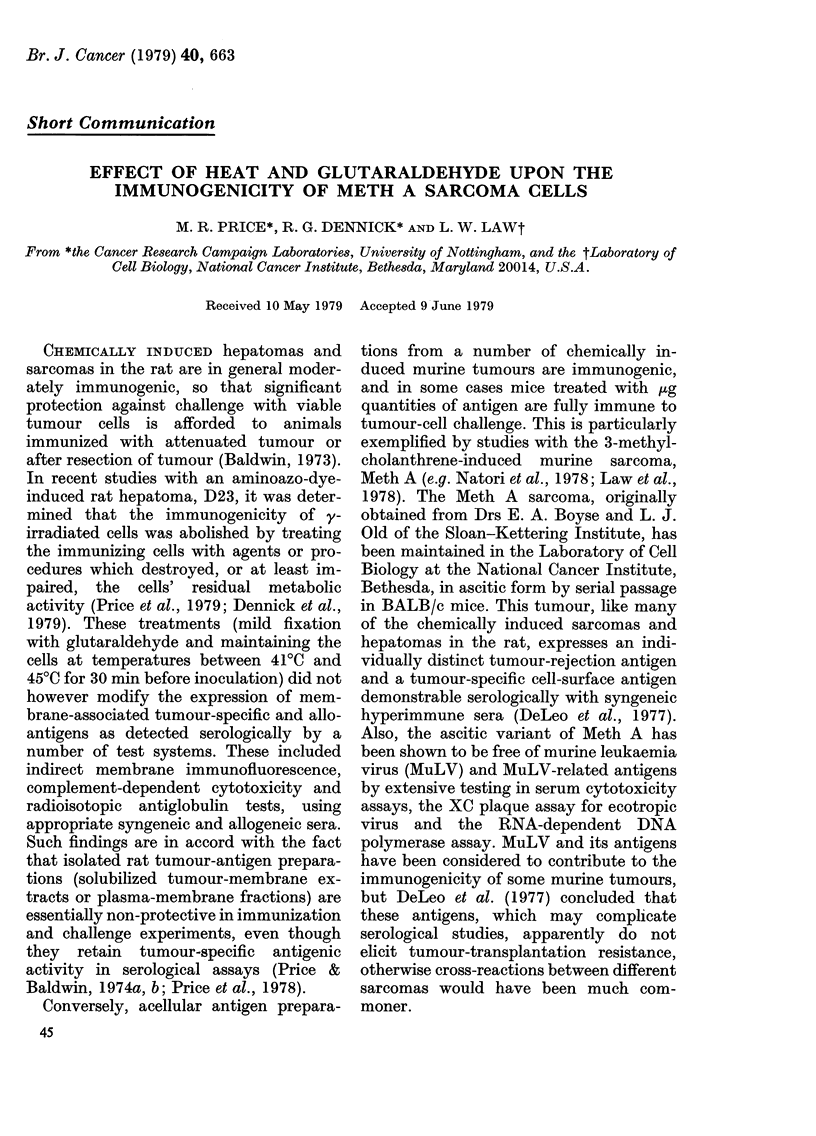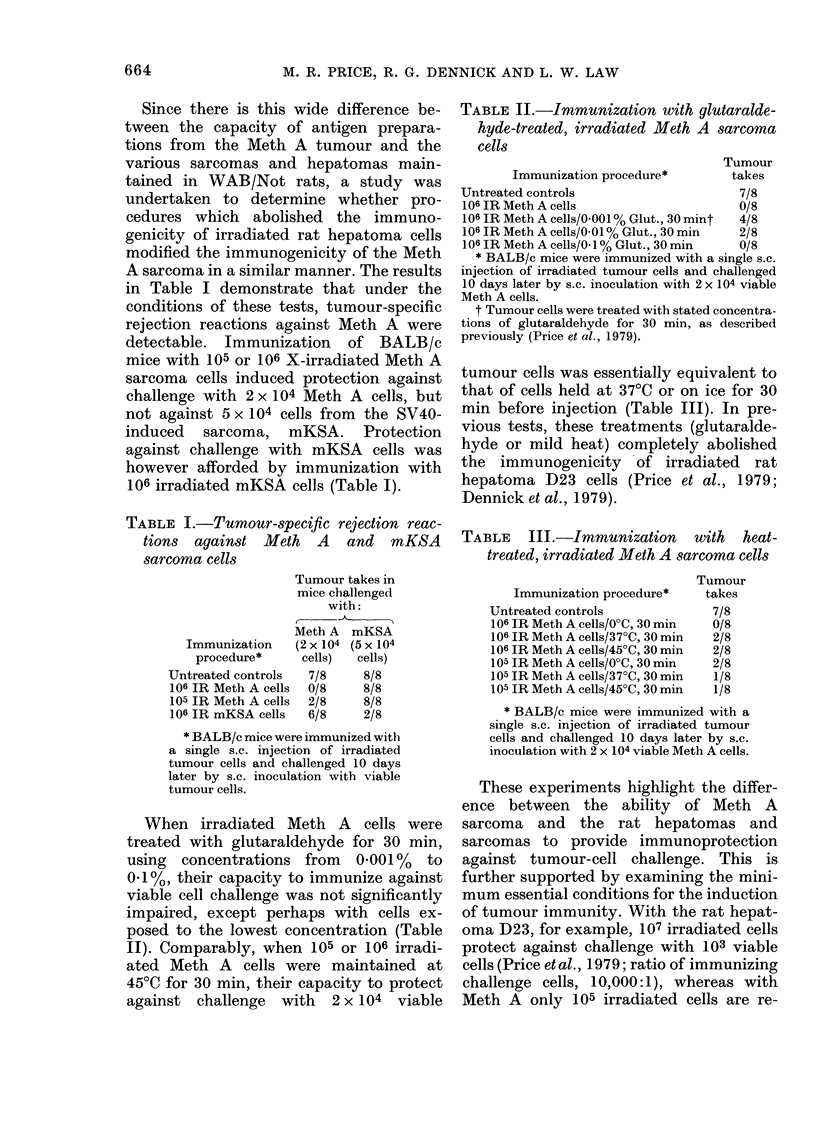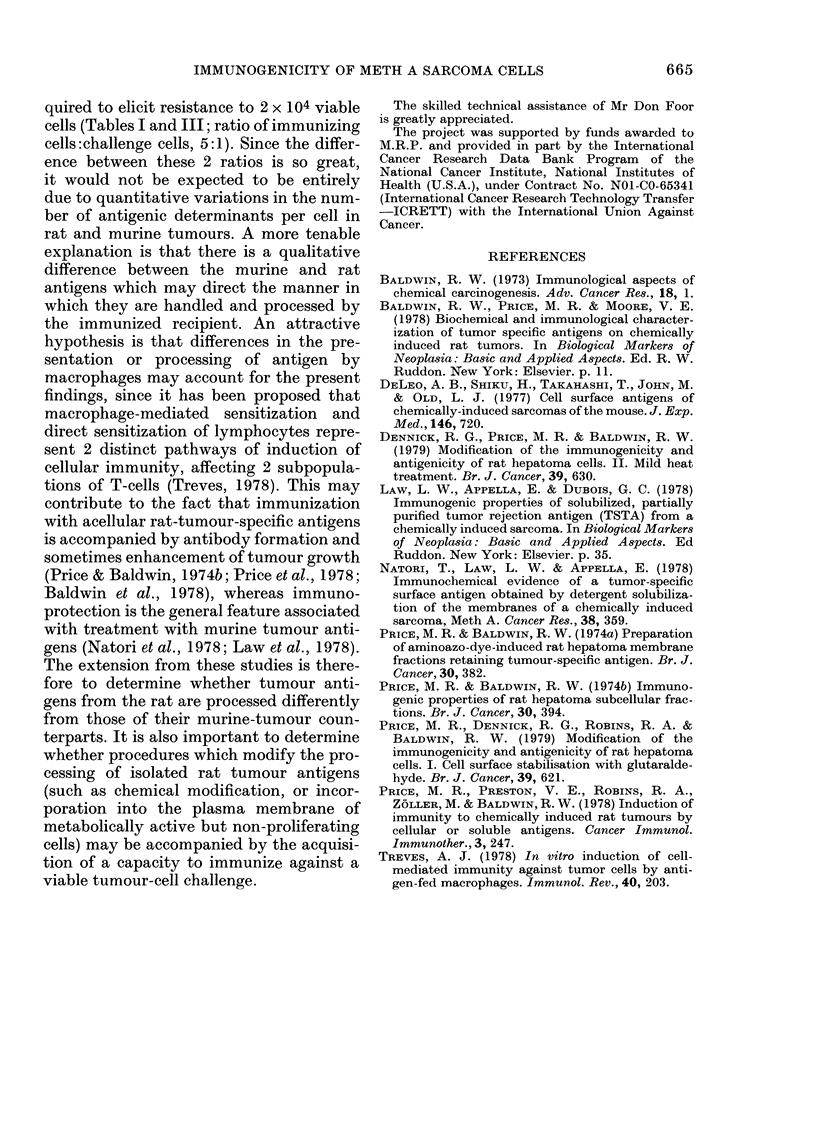# Effect of heat and glutaraldehyde upon the immunogenicity of Meth A sarcoma cells.

**DOI:** 10.1038/bjc.1979.234

**Published:** 1979-10

**Authors:** M. R. Price, R. G. Dennick, L. W. Law


Br. J. Cancer (1979) 40, 663

### Short Communication

### EFFECT OF HEAT AND GLUTARALDEHYDE UPON THE IMMUNOGENICITY OF METH A SARCOMA CELLS

M. R. PRICE*, R. G. DENNICK* AND L. W. LAW†

*From *the Cancer Research Campaign Laboratories, University of Nottingham, and the †Laboratory of Cell Biology, National Cancer Institute, Bethesda, Maryland 20014, U.S.A.*

Received 10 May 1979 Accepted 9 June 1979

CHEMICALLY INDUCED hepatomas and sarcomas in the rat are in general moderately immunogenic, so that significant protection against challenge with viable tumour cells is afforded to animals immunized with attenuated tumour or after resection of tumour (Baldwin, 1973). In recent studies with an aminoazo-dye-induced rat hepatoma, D23, it was determined that the immunogenicity of $\gamma$-irradiated cells was abolished by treating the immunizing cells with agents or procedures which destroyed, or at least impaired, the cells' residual metabolic activity (Price *et al.*, 1979; Dennick *et al.*, 1979). These treatments (mild fixation with glutaraldehyde and maintaining the cells at temperatures between 41°C and 45°C for 30 min before inoculation) did not however modify the expression of membrane-associated tumour-specific and allo-antigens as detected serologically by a number of test systems. These included indirect membrane immunofluorescence, complement-dependent cytotoxicity and radioisotopic antiglobulin tests, using appropriate syngeneic and allogeneic sera. Such findings are in accord with the fact that isolated rat tumour-antigen preparations (solubilized tumour-membrane extracts or plasma-membrane fractions) are essentially non-protective in immunization and challenge experiments, even though they retain tumour-specific antigenic activity in serological assays (Price & Baldwin, 1974*a*, *b*; Price *et al.*, 1978).

Conversely, acellular antigen preparations from a number of chemically induced murine tumours are immunogenic, and in some cases mice treated with $\mu$g quantities of antigen are fully immune to tumour-cell challenge. This is particularly exemplified by studies with the 3-methylcholanthrene-induced murine sarcoma, Meth A (*e.g.* Natori *et al.*, 1978; Law *et al.*, 1978). The Meth A sarcoma, originally obtained from Drs E. A. Boyse and L. J. Old of the Sloan–Kettering Institute, has been maintained in the Laboratory of Cell Biology at the National Cancer Institute, Bethesda, in ascitic form by serial passage in BALB/c mice. This tumour, like many of the chemically induced sarcomas and hepatomas in the rat, expresses an individually distinct tumour-rejection antigen and a tumour-specific cell-surface antigen demonstrable serologically with syngeneic hyperimmune sera (DeLeo *et al.*, 1977). Also, the ascitic variant of Meth A has been shown to be free of murine leukaemia virus (MuLV) and MuLV-related antigens by extensive testing in serum cytotoxicity assays, the XC plaque assay for ecotropic virus and the RNA-dependent DNA polymerase assay. MuLV and its antigens have been considered to contribute to the immunogenicity of some murine tumours, but DeLeo *et al.* (1977) concluded that these antigens, which may complicate serological studies, apparently do not elicit tumour-transplantation resistance, otherwise cross-reactions between different sarcomas would have been much commoner.

Since there is this wide difference between the capacity of antigen preparations from the Meth A tumour and the various sarcomas and hepatomas maintained in WAB/Not rats, a study was undertaken to determine whether procedures which abolished the immunogenicity of irradiated rat hepatoma cells modified the immunogenicity of the Meth A sarcoma in a similar manner. The results in Table I demonstrate that under the conditions of these tests, tumour-specific rejection reactions against Meth A were detectable. Immunization of BALB/c mice with $10^5$ or $10^6$ X-irradiated Meth A sarcoma cells induced protection against challenge with $2 \times 10^4$ Meth A cells, but not against $5 \times 10^4$ cells from the SV40-induced sarcoma, mKSA. Protection against challenge with mKSA cells was however afforded by immunization with $10^6$ irradiated mKSA cells (Table I).

TABLE I.—*Tumour-specific rejection reactions against Meth A and mKSA sarcoma cells*

| Immunization procedure* | Tumour takes in mice challenged with: Meth A ($2 \times 10^4$ cells) | mKSA ($5 \times 10^4$ cells) |
|---|---|---|
| Untreated controls | 7/8 | 8/8 |
| $10^6$ IR Meth A cells | 0/8 | 8/8 |
| $10^5$ IR Meth A cells | 2/8 | 8/8 |
| $10^6$ IR mKSA cells | 6/8 | 2/8 |

* BALB/c mice were immunized with a single s.c. injection of irradiated tumour cells and challenged 10 days later by s.c. inoculation with viable tumour cells.

When irradiated Meth A cells were treated with glutaraldehyde for 30 min, using concentrations from 0·001% to 0·1%, their capacity to immunize against viable cell challenge was not significantly impaired, except perhaps with cells exposed to the lowest concentration (Table II). Comparably, when $10^5$ or $10^6$ irradiated Meth A cells were maintained at 45°C for 30 min, their capacity to protect against challenge with $2 \times 10^4$ viable tumour cells was essentially equivalent to that of cells held at 37°C or on ice for 30 min before injection (Table III). In previous tests, these treatments (glutaraldehyde or mild heat) completely abolished the immunogenicity of irradiated rat hepatoma D23 cells (Price *et al.*, 1979; Dennick *et al.*, 1979).

TABLE II.—*Immunization with glutaraldehyde-treated, irradiated Meth A sarcoma cells*

| Immunization procedure* | Tumour takes |
|---|---|
| Untreated controls | 7/8 |
| $10^6$ IR Meth A cells | 0/8 |
| $10^6$ IR Meth A cells/0·001% Glut., 30 min† | 4/8 |
| $10^6$ IR Meth A cells/0·01% Glut., 30 min | 2/8 |
| $10^6$ IR Meth A cells/0·1% Glut., 30 min | 0/8 |

* BALB/c mice were immunized with a single s.c. injection of irradiated tumour cells and challenged 10 days later by s.c. inoculation with $2 \times 10^4$ viable Meth A cells.

† Tumour cells were treated with stated concentrations of glutaraldehyde for 30 min, as described previously (Price *et al.*, 1979).

TABLE III.—*Immunization with heat-treated, irradiated Meth A sarcoma cells*

| Immunization procedure* | Tumour takes |
|---|---|
| Untreated controls | 7/8 |
| $10^6$ IR Meth A cells/0°C, 30 min | 0/8 |
| $10^6$ IR Meth A cells/37°C, 30 min | 2/8 |
| $10^6$ IR Meth A cells/45°C, 30 min | 2/8 |
| $10^5$ IR Meth A cells/0°C, 30 min | 2/8 |
| $10^5$ IR Meth A cells/37°C, 30 min | 1/8 |
| $10^5$ IR Meth A cells/45°C, 30 min | 1/8 |

* BALB/c mice were immunized with a single s.c. injection of irradiated tumour cells and challenged 10 days later by s.c. inoculation with $2 \times 10^4$ viable Meth A cells.

These experiments highlight the difference between the ability of Meth A sarcoma and the rat hepatomas and sarcomas to provide immunoprotection against tumour-cell challenge. This is further supported by examining the minimum essential conditions for the induction of tumour immunity. With the rat hepatoma D23, for example, $10^7$ irradiated cells protect against challenge with $10^3$ viable cells (Price *et al.*, 1979; ratio of immunizing challenge cells, 10,000:1), whereas with Meth A only $10^5$ irradiated cells are required to elicit resistance to $2 \times 10^4$ viable cells (Tables I and III; ratio of immunizing cells:challenge cells, 5:1). Since the difference between these 2 ratios is so great, it would not be expected to be entirely due to quantitative variations in the number of antigenic determinants per cell in rat and murine tumours. A more tenable explanation is that there is a qualitative difference between the murine and rat antigens which may direct the manner in which they are handled and processed by the immunized recipient. An attractive hypothesis is that differences in the presentation or processing of antigen by macrophages may account for the present findings, since it has been proposed that macrophage-mediated sensitization and direct sensitization of lymphocytes represent 2 distinct pathways of induction of cellular immunity, affecting 2 subpopulations of T-cells (Treves, 1978). This may contribute to the fact that immunization with acellular rat-tumour-specific antigens is accompanied by antibody formation and sometimes enhancement of tumour growth (Price & Baldwin, 1974*b*; Price *et al.*, 1978; Baldwin *et al.*, 1978), whereas immunoprotection is the general feature associated with treatment with murine tumour antigens (Natori *et al.*, 1978; Law *et al.*, 1978). The extension from these studies is therefore to determine whether tumour antigens from the rat are processed differently from those of their murine-tumour counterparts. It is also important to determine whether procedures which modify the processing of isolated rat tumour antigens (such as chemical modification, or incorporation into the plasma membrane of metabolically active but non-proliferating cells) may be accompanied by the acquisition of a capacity to immunize against a viable tumour-cell challenge.

The skilled technical assistance of Mr Don Foor is greatly appreciated.

The project was supported by funds awarded to M.R.P. and provided in part by the International Cancer Research Data Bank Program of the National Cancer Institute, National Institutes of Health (U.S.A.), under Contract No. N01-C0-65341 (International Cancer Research Technology Transfer —ICRETT) with the International Union Against Cancer.

### REFERENCES

Baldwin, R. W. (1973) Immunological aspects of chemical carcinogenesis. *Adv. Cancer Res.*, **18**, 1.

Baldwin, R. W., Price, M. R. & Moore, V. E. (1978) Biochemical and immunological characterization of tumor specific antigens on chemically induced rat tumors. In *Biological Markers of Neoplasia: Basic and Applied Aspects.* Ed. R. W. Ruddon. New York: Elsevier. p. 11.

DeLeo, A. B., Shiku, H., Takahashi, T., John, M. & Old, L. J. (1977) Cell surface antigens of chemically-induced sarcomas of the mouse. *J. Exp. Med.*, **146**, 720.

Dennick, R. G., Price, M. R. & Baldwin, R. W. (1979) Modification of the immunogenicity and antigenicity of rat hepatoma cells. II. Mild heat treatment. *Br. J. Cancer*, **39**, 630.

Law, L. W., Appella, E. & Dubois, G. C. (1978) Immunogenic properties of solubilized, partially purified tumor rejection antigen (TSTA) from a chemically induced sarcoma. In *Biological Markers of Neoplasia: Basic and Applied Aspects.* Ed Ruddon. New York: Elsevier. p. 35.

Natori, T., Law, L. W. & Appella, E. (1978) Immunochemical evidence of a tumor-specific surface antigen obtained by detergent solubilization of the membranes of a chemically induced sarcoma, Meth A. *Cancer Res.*, **38**, 359.

Price, M. R. & Baldwin, R. W. (1974*a*) Preparation of aminoazo-dye-induced rat hepatoma membrane fractions retaining tumour-specific antigen. *Br. J. Cancer*, **30**, 382.

Price, M. R. & Baldwin, R. W. (1974*b*) Immunogenic properties of rat hepatoma subcellular fractions. *Br. J. Cancer*, **30**, 394.

Price, M. R., Dennick, R. G., Robins, R. A. & Baldwin, R. W. (1979) Modification of the immunogenicity and antigenicity of rat hepatoma cells. I. Cell surface stabilisation with glutaraldehyde. *Br. J. Cancer*, **39**, 621.

Price, M. R., Preston, V. E., Robins, R. A., Zöller, M. & Baldwin, R. W. (1978) Induction of immunity to chemically induced rat tumours by cellular or soluble antigens. *Cancer Immunol. Immunother.*, **3**, 247.

Treves, A. J. (1978) *In vitro* induction of cell-mediated immunity against tumor cells by antigen-fed macrophages. *Immunol. Rev.*, **40**, 203.